# Effectiveness of trans-nasal humidified rapid insufflation ventilatory exchange compared with standard facemask oxygenation for pre- and apneic oxygenation during anesthesia induction: A meta-analysis based on randomized controlled trials

**DOI:** 10.1371/journal.pone.0302626

**Published:** 2024-04-30

**Authors:** Delai Zheng, Yi Yao, Chuan Luo, Yanhui Yang, Bogang Chen, Nana Li, Yi Wang

**Affiliations:** 1 Department of Cardiothoracic Surgery, The First People’s Hospital of Neijiang, Neijiang Affiliated Hospital of Chongqing Medical University, Neijiang, Sichuan, China; 2 Department of Anesthesiology, The First People’s Hospital of Neijiang, Neijiang Affiliated Hospital of Chongqing Medical University, Neijiang, Sichuan, China; Acibadem Maslak Hospital: Acibadem Maslak Hastanesi, TURKEY

## Abstract

**Purpose:**

To further identify the effectiveness of trans-nasal humidified rapid insufflation ventilatory exchange (THRIVE) for pre- and apneic oxygenation during the anesthesia induction by comparison to facemask ventilation (FMV) based on current available evidence.

**Methods:**

Medline, EMBASE, Web of Science, Cochrane Library and CNKI databases were searched from inception to December 22, 2023 for available randomized controlled trials (RCTs). Primary outcomes were PaO_2_ and PaCO_2_ after intubation and safe apnoea time. Secondary outcomes included the O_2_ desaturation, end expiratory carbon dioxide (EtCO_2_) and complications. The effect measures for continuous and categorical outcomes were separately the mean difference (MD) and relative risk (RR) with 95% confidence interval.

**Results:**

Twelve RCTs with 403 patients in the THRIVE group and 401 patients in th FMV group were included. Pooled results demonstrated that the PaO_2_ after intubation was significantly higher (MD = 82.90mmHg, 95% CI: 12.25~153.54mmHg, P = 0.02) and safe apnoea time (MD = 103.81s, 95% CI: 42.07~165.56s, P = 0.001) was longer in the THRIVE group. Besides, the incidence rate of O_2_ desaturation (RR = 0.28, 95% CI: 0.12–0.66, P = 0.004) and gastric insufflation (RR = 0.26, 95% CI: 0.13–0.49, P<0.001) was significantly lower in the THRIVE group.

**Conclusion:**

Based on current evidence, THRIVE manifested better effectiveness representing as improved oxygenation, prolonged safe apnoea time and decreased risk of complications compared to standard FMV in surgical patients. Therefore, THRIVE could be served as a novel and valuable oxygenation technology for patients during anesthesia induction.

## Introduction

In clinics, face mask ventilation (FMV) during anesthesia induction is a common airway management method, but it also has some drawbacks and limitations. When applying FMV, improper airway management may lead to the entry of gas into the stomach, increasing gastric volume and thereby elevating the risk of gastric distention and aspiration for the patient [1]. Excessive airway pressure during FMV may cause airway injury, especially in patients who require careful handling, such as children and the elderly [2]. Some patients may have difficulty obtaining an effective seal due to facial abnormalities or other reasons, affecting the effectiveness of FMV. Additionally, accurately controlling tidal volume during FMV can be relatively challenging, potentially resulting in inadequate or excessive tidal volumes [3, 4]. Inserting a nasogastric tube may be relatively difficult when using FMV, which could impact the intraoperative need for gastric emptying. Implementing positive end-expiratory pressure (PEEP) during FMV may also be relatively challenging, and PEEP may be necessary for certain patients, such as those with acute respiratory distress syndrome (ARDS) [5, 6].

Trans-nasal humidified rapid insufflation ventilatory exchange (THRIVE), also known as high-flow nasal cannula (HFNC) oxygen therapy, is a novel oxygen therapy method. It delivers a specific concentration of high-flow air-oxygen mixture directly to the patient through heating and humidification, eliminating the need for a closed nasal catheter [7]. THRIVE provides heated and humidified gas at a maximum flow rate of 70 L/min, maintaining stable FiO_2_ for the patient while reducing anatomical dead space in the upper airway and increasing intratracheal oxygen concentration. Studies have shown that continuous THRIVE can alleviate the patient’s respiratory effort and produce a continuous positive end-expiratory pressure (PEEP) effect [8]. The positive pressure effect increases with the rising flow rate, providing 1 cm H_2_O PEEP with a closed mouth and 0.5 cm H_2_O PEEP with an open mouth for every 10 L/min increase in flow rate [8]. The positive pressure effect opens the patient’s upper airway, reduces intrapulmonary shunts, and further enhances the patient’s oxygen reserve [9]. However, currently, there is no high-quality evidence elucidating the specific effectiveness of THRIVE during anesthesia induction.

Therefore, this meta-analysis aimed to further determine the effectiveness of THRIVE for pre- and apneic oxygenation during the anesthesia induction by comparison with FMV based on available randomized controlled trials (RCTs), which might contribute to the clinical application of THRIVE.

## Materials and methods

This meta-analysis was performed according to the Preferred Reporting Items for Systematic Review and Meta-Analyses 2020 [10].

### Ethical approval

All procedures performed in studies involving human participants were in accordance with the ethical standards of the institutional and/or national research committee and with the 1964 Helsinki Declaration and its later amendments or comparable ethical standards. For this type of study, formal consent is not needed.

### Literature search

In this meta-analysis, Medline, EMBASE, Web of Science, Cochrane Library and CNKI databases were searched from their inception up to December 22, 2023. Following terms were applied during the search: transnasal humidified rapid-insufflation ventilatory exchange, THRIVE, surgery, operation, randomized, randomly and random. The specific literature search strategy was as follows: (transnasal humidified rapid-insufflation ventilatory exchange OR THRIVE) AND (surgery OR operation) AND (randomized OR randomly OR random). MeSH terms and free texts were applied during the search. Besides, references listed in included studies were also reviewed.

### Inclusion criteria

Studies met following criteria were included: 1) RCTs comparing the effectiveness of THRIVE and FMV during the anesthesia induction; 2) at least one of following indicators was involved with relevant data: PaO_2_ and PaCO_2_ after intubation, safe apnoea time, O_2_ desaturation, end expiratory carbon dioxide (EtCO_2_) and complications such as gastric insufflation, reflux, arrhythmias, nausea and vomiting; 3) adult patients (≥ 18-year-old).

### Exclusion criteria

Studies met following criteria were excluded: 1) the intervention for control group was not the FMV, including the bite block or nasal cannula ventilation; 2) full texts were not available; 3) letters, editorials, meeting abstracts, animal trials, meta-analyses or reviews; 4) duplicated or overlapped data.

### Data collection

We extracted following data from each included RCTs: the first author, publication year, sample size of each group, country, type of surgery, intervention in THRIVE group and FMV group, endpoints including the PaO_2_ and PaCO_2_ after intubation, safe apnoea time, O_2_ desaturation, EtCO_2_ and complications and corresponding data.

For the safe apnoea time, the definitions of included studies differ. Three studies defined apnoea time from the onset of cessation of breathing until the SpO_2_ decreased to 95% or the apnoea time reached 5 or 6 min [11–13]. Ding et al. defined apnoea time from the end of intravenous muscle relaxation until the SpO_2_ decreased to 94% [14]. In the study by Mir et al., it was defined as from the cessation of spontaneous breathing until the SpO_2_ decreased to 90% [15]. While six RCTs explored the impact of THRIVE on deoxysaturation [11, 15–19], only two studies were valid for the analysis [16, 17]. The deoxysaturation was separately defined as SpO_2_≤90% [17] and SpO_2_≤93% [16] in these two studies.

### Methodological quality assessment

The risk of bias in included RCTs was assessed by the Review Manager software with the guidance of the Cochrane Collaboration risk-of-bias tool [20]. Studies were divided into high, low or unclear risk of bias involving the aspects including the selection, performance, detection, attrition, reporting and others.

The literature search, selection, data collection and methodological quality assessment were all performed by two authors independently. Any disagreement was resolved by discussion.

### Statistical analysis

In this meta-analysis, all analysis was performed by Review Manager (RevMan version 5.4.1). The statistical method for continuous variables was inverse variance and the effect measure was mean difference (MD). The statistical method for categorical variables was Mantel-Haenszel and the effect measure was relative risk (RR). Heterogeneity between studies was evaluated by the I^2^ statistic and Q test. When significant heterogeneity was observed representing as I^2^ > 50% and/or P < 0.10, the random-effect mode was applied; otherwise, the fixed-effect model was used [21, 22]. For studies providing data in median and range or interquartile range, the method described by Wan et al. was used to convert them into mean and standard deviation (SD) [23].

## Results

### Literature search process

**Fig 1:**
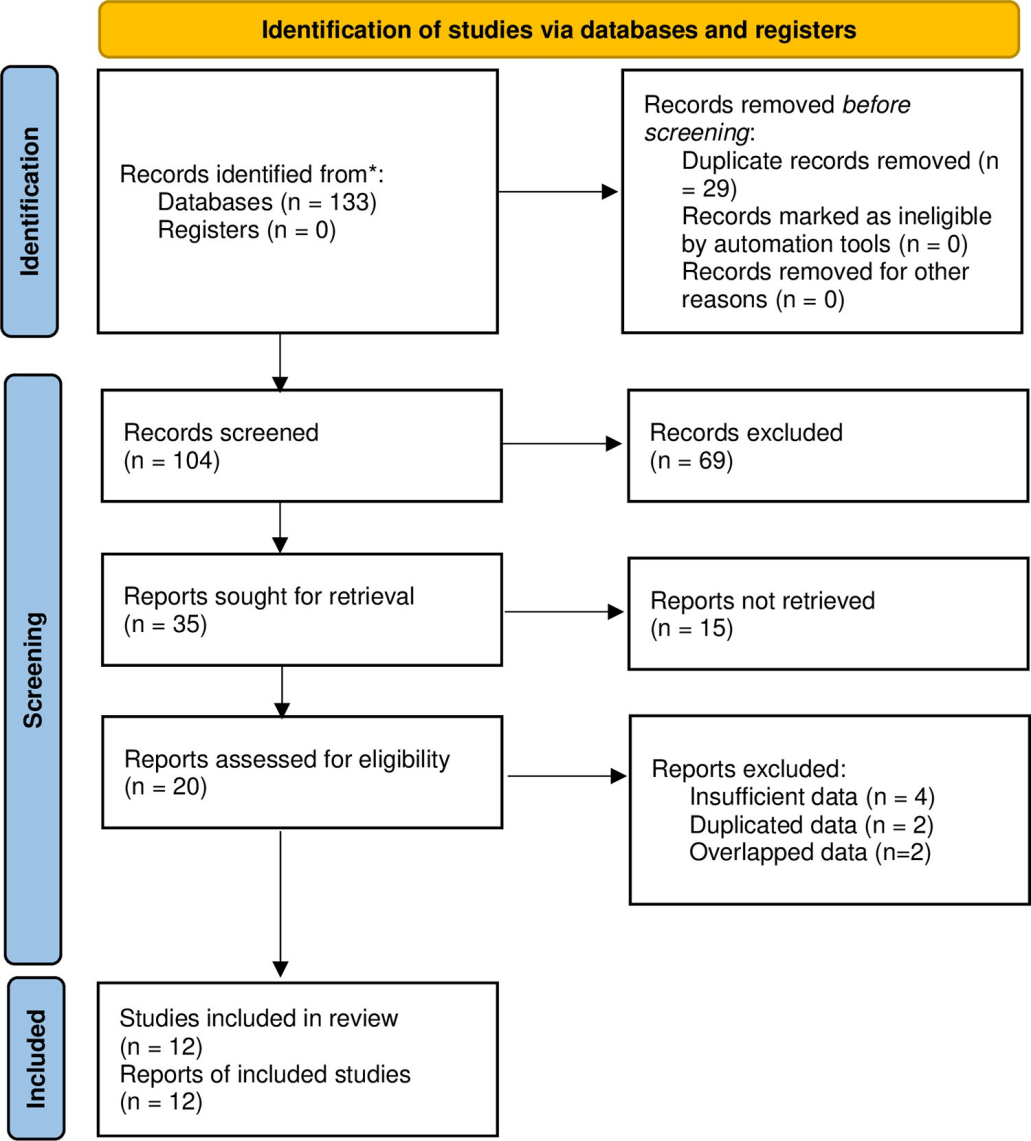
Prisma flow diagram of this meta-analysis.

One hundred and thirty-three records were identified from the five databases. Detailed literature selection process for this meta-analysis was presented in **Fig 1**. Eventually, 12 RCTs were included in our analysis [11–19, 24–26].

### Basic characteristics of included RCTs

**Fig 2:**
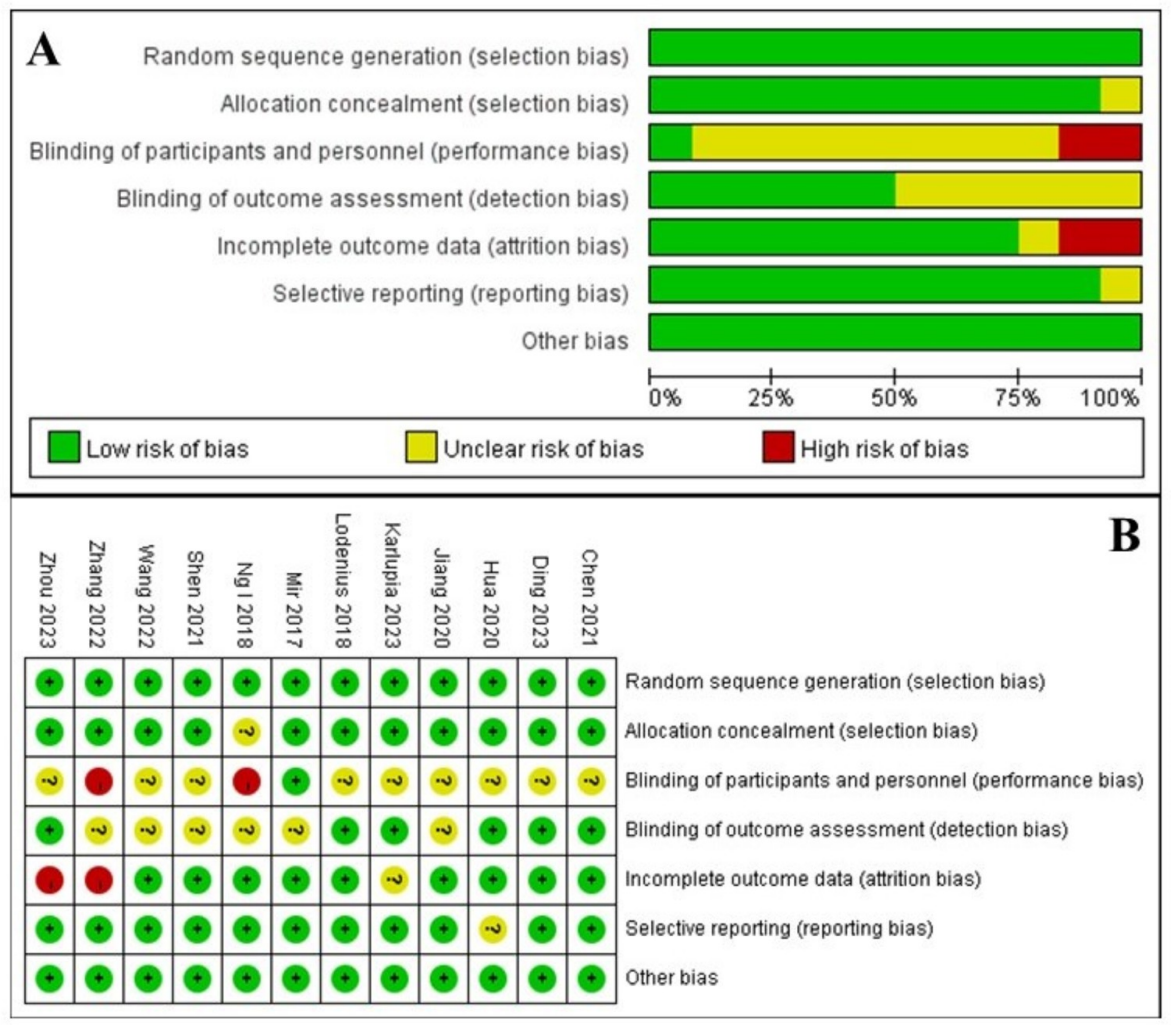
Risk of bias assessment. A. Risk of bias summary. B. Risk of bias graph. The plus sign indicates low risk, the sinus sign indicates high risk, and the question sign mark uncertain risk.

A total of 804 cases were enrolled with 403 patients in the THRIVE group and 401 patients in the FMV group. Specific information was presented in Table 1. The methodological quality assessment was shown in **Fig 2**, which indicated the high-quality of included RCTs.

**Table 1:** Basic characteristics of included studies.

Author	Year	Sample size (THRIVE/FMV)	Country	Type of surgery	Intervention in THRIVE group	Intervention in FMV group	Endpoints
Mir [15]	2017	20/20	British	Emergency surgery	30-70L/min	12L/min	①②③④
Lodenius [16]	2018	40/39	Sweden	Emergency surgery	40-70L/min	10L/min	④⑤
Ng I [18]	2018	24/24	Australia	GA in neurosurgical patients	30-50L/min	10L/min	④
Hua [17]	2020	30/28	China	GA in elderly	30-70L/min	10L/min	④⑥
Jiang [24]	2020	30/30	China	Cholecystectomy in obese patients	30-70L/min	10L/min	①②⑥
Chen [11]	2021	20/20	China	Uvulopharyngopalatoplasty	10-70L/min	6-10L/min	①②③④
Shen [19]	2021	25/25	China	Lobectomy	30-70L/min	8L/min	①②④
Wang [12]	2022	20/20	China	Gastrointestinal surgery in elderly	40L/min	6L/min	①②③⑤
Zhang [13]	2022	25/25	China	GA in elderly	60L/min	10L/min	③⑤
Karlupia [25]	2023	40/40	India	Emergency surgery	60L/min	12L/min	①②
Ding [14]	2023	29/28	China	Cholecystectomy	30-70L/min	6L/min	①②③⑤⑥
Zhou [26]	2023	100/102	China	GA	60L/min	12L/min	④⑥

THRIVE: trans-nasal humidified rapid insufflation ventilatory exchange; FMV: face mask ventilation; GA: general anesthesia; ①PaO_2_(after intubation), ②PaCO_2_ (after intubation), ③Safe apnoea time, ④O_2_ desaturation, ⑤EtCO_2_: end expiratory carbon dioxide; ⑥Complication

### Meta-analysis results of primary outcomes

**Fig 3:**
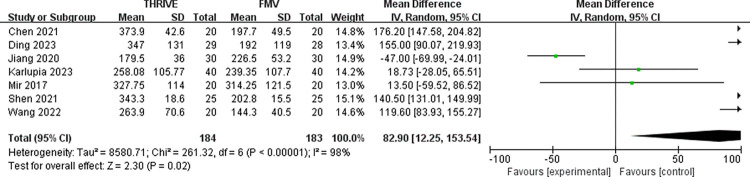
Forest plot for PaO_2_ in THRIVE versus FMV after intubation. THRIVE: trans-nasal humidified rapid insufflation ventilatory exchange; FMV: face mask ventilation.

Seven RCTs explored the effect of THRIVE on the PaO_2_ and PaCO_2_ after intubation with a total of 367 participants [11, 12, 14, 15, 19, 24, 25]. Pooled results demonstrated that the PaO_2_ in the THRIVE group was significantly higher than that in the FMV group (MD = 82.90mmHg, 95% CI: 12.25~153.54mmHg, P = 0.02; I^2^ = 98%, P<0.001) (**Fig 3**). However, no significant statistical difference of PaCO_2_ between the two groups was observed (MD = 2.05mmHg, 95% CI: -0.48~4.58mmHg, P = 0.11; I^2^ = 75%, P<0.001). (**Table 2**)

**Table 2:** Results of meta-analysis.

	No. of studies	Mean difference	95% confidence interval	P value	I^2^	P value for heterogeneity
Primary outcomes						
PaO_2_ after intubation, mmHg	7 [11, 12, 14, 15, 19, 24, 25]	82.90	12.25~153.54	0.02	98%	<0.001
PaCO_2_ after intubation, mmHg	7 [11, 12, 14, 15, 19, 24, 25]	2.05	-0.48~4.58	0.11	75%	<0.001
Safe apnoea time, s	5 [11–15]	103.81	42.07~165.56	0.001	95%	<0.001
Secondary outcomes						
O_2_ desaturation, n	6 [11, 15–19]	0.28 (RR)	0.12–0.66	0.004	0%	0.34
EtCO_2_, mmHg	4 [12–14, 16]	3.54	-0.70~7.78	0.10	91%	<0.001
Complications, n						
Gastric insufflation	2 [14, 26]	0.26 (RR)	0.13~0.49	<0.001	0%	0.67
Reflux	1 [14]	0.24 (RR)	0.06~1.02	0.05	-	-
Arrhythmias	1 [17]	1.50 (RR)	0.38–6.00	0.57	-	-
Nausea and vomiting	1 [24]	0.22 (RR)	0.02~2.14	0.19	-	-

RR: relative risk; EtCO_2_: end expiratory carbon dioxide.

**Fig 4:**
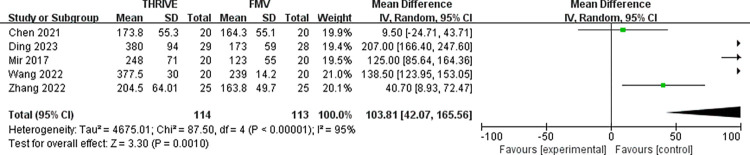
Forest plot for safe apnoea time in THRIVE versus FMV after intubation. THRIVE: trans-nasal humidified rapid insufflation ventilatory exchange; FMV: face mask ventilation.

Five RCTs involving with 227 patients identified the effect of THRIVE on the safe apnoea time [11–15]. Pooled results indicated that the THRIVE could significantly increase the safe apnoea time compared with FMV during the anesthesia induction (MD = 103.81s, 95% CI: 42.07~165.56s, P = 0.001; I^2^ = 95%, P<0.001) (**Fig 4**). (**Table 2**)

### Meta-analysis results of secondary outcomes

**Fig 5:**
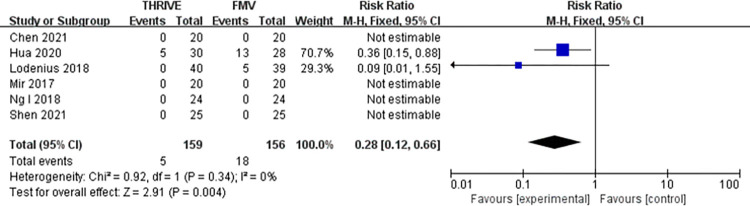
Forest plot for O_2_ desaturation in THRIVE versus FMV after intubation. THRIVE: trans-nasal humidified rapid insufflation ventilatory exchange; FMV: face mask ventilation.

Six studies with 315 patients investigated the incidence of O_2_ desaturation [11, 15–19]. However, only the data of two studies was estimable [16, 17]. Pooled results manifested that THRIVE significantly decreased the risk of O_2_ desaturation (RR = 0.28, 95% 95% CI: 0.12–0.66, P = 0.004; I^2^ = 0%, P = 0.34) (**Fig 5**).

Besides, four studies manifested the effect of THRIVE on the EtCO_2_ [12–14, 16]. However, no significant statistical difference was observed between the THRIVE and FMV groups (MD = 3.54mmHg, 95% CI: -0.70~7.78mmHg, P = 0.10; I^2^ = 91%, P<0.001). (**Table 2**)

**Fig 6:**
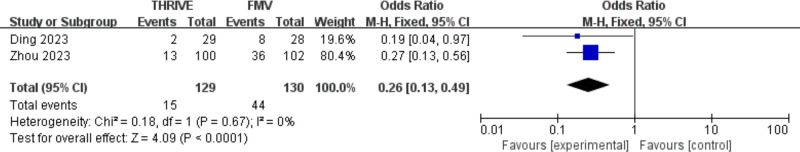
Forest plot for gastric insufflation in THRIVE versus FMV after intubation. THRIVE: trans-nasal humidified rapid insufflation ventilatory exchange; FMV: face mask ventilation.

As for the complications, the incidence rate of gastric insufflation in the THRIVE group was significantly lower than that in the FMV group (RR = 0.26, 95% 95% CI: 0.13–0.49, P<0.001; I^2^ = 0%, P = 0.67) based on the data from two available RCTs (**Fig 6**) [14, 26]. The incidence risk for reflux (RR = 0.24, 95% CI: 0.06–1.02, P = 0.05), arrhythmias (RR = 1.50, 95% CI: 0.38–6.00, P = 0.57), nausea and vomiting (RR = 0.22, 95% CI: 0.02–2.14, P = 0.19) was not statistically difference between the two groups (**Table 2**).

## Discussion

In recent years, THRIVE, as a novel method of "apneic oxygenation," has gradually gained attention and application in clinical practice. "Apneic oxygenation" refers to the administration of high concentrations of oxygen through the airway when the body is not in respiratory motion, promoting the exchange of oxygen in the alveoli [27, 28]. Generally, the maximum flow of dry oxygen that can be inhaled by awake patients is limited to 15 L/min, as excessive flow can lead to discomfort such as dry nasal mucosa and frontal/sinus pain. THRIVE, on the other hand, can increase the maximum flow to 70 mL/min through a heating and humidifying device [29]. In theory, at this flow rate, anesthesia patients’ oxygenation can be maintained, achieving CO_2_ clearance and "diffusive oxygenation". Compared to traditional oxygen therapy methods, THRIVE, by setting the gas flow higher than the patient’s inspiratory peak flow, can provide a stable inspired oxygen concentration and low-level positive pressure. This reduces nasopharyngeal dead space, improves airway mucosal clearance function, and to some extent, has the effect of opening alveoli and promoting ventilation [30]. Additionally, THRIVE can establish an O_2_ pressure difference gradient between the upper airway and alveoli, as well as between alveoli and pulmonary capillaries, driving O_2_ diffusion into the pulmonary capillaries, achieving the "apneic diffusive oxygenation" effect [31]. Currently, THRIVE is primarily used for respiratory support in patients with respiratory dysfunction to improve and treat hypercapnia or mild to moderate hypoxic respiratory failure [32–34]. However, its application in the field of anesthesia remains limited.

According to the results of our meta-analysis, THRIVE technique could extend the safe apnea time during patient anesthesia induction, increase oxygen reserve, reduce the risk of hypoxemia during endotracheal intubation, and also decrease the risk of gastric distention. However, in our analysis, THRIVE did not demonstrate an obvious advantage in improving carbon dioxide retention, and further exploration is needed in future studies.

As thoracic surgeons, we paid particular attention to the research conducted by Shen and colleagues [19]. Their results demonstrated that THRIVE played a role in reducing oxygen pressure decline and improving the safety of endotracheal intubation among patients undergoing lung lobectomy with the need for double-lumen endotracheal intubation [19]. During the insertion of a double-lumen endotracheal tube, ventilation can be maintained through THRIVE to improve patient oxygenation. While adjustments are made with a fiberoptic bronchoscope after the placement of the double-lumen endotracheal tube, ventilation can be connected to the anesthesia machine. However, there is still a risk of hypoxia in case of poor alignment and inadequate ventilation. THRIVE significantly extends the safe apnea time, providing a time buffer for the adjustment of the tube position.

Notably, in this meta-analysis, we identified the significant effect of THRIVE on the decreased risk of gastric insufflation. Although the association of THRIVE with decreased risk of reflux did not reach the statistical difference, a clear trend that THRIVE reduced the incidence of reflux was observed (RR = 0.24, P = 0.05). Therefore, we deem that THRIVE might also decrease the incidence rate of reflux among surgical patients. However, more RCTs are still needed to verify this issue.

THRIVE still has some clinical value after the start of surgery. First, THRIVE can continuously provide oxygen to patients during surgery, ensuring the maintenance of their oxygen levels throughout the procedure. This is particularly crucial for procedures with longer durations. Second, during the surgical process, especially during anesthesia induction and maintenance stages, the use of THRIVE can reduce the risk of patients experiencing hypoxemia, enhancing the safety of anesthesia. Third, by delivering humidified gas, THRIVE helps support airway humidification, reducing dryness of the respiratory mucosa and improving airway conditions. Fourth, for certain specialized surgeries like head and neck procedures, THRIVE can maintain patient oxygenation during surgery, minimizing interference with ventilation. Fifth, the use of THRIVE may contribute to reducing the need for mechanical ventilation during surgery, providing a more natural form of ventilatory support.

There are several limitations in our meta-analysis. First, the overall sample size was relatively small and most RCTs were from China. Second, a number of confounding parameters exist in our analysis such as the type of surgery and intervention. More detailed analysis was needed to further verify our conclusions. Third, obvious heterogeneity was detected during the analysis of some indicators such as the PaO_2_ after intubation, safe apnoea time and EtCO_2,_ we were unable to conduct more analysis to identify the source of heterogeneity due to the limited available data. Four, in most included studies, the basic conditions of patients, including combined lung diseases, were not mentioned. Therefore, further exploration is needed to determine whether THRIVE is practical for all patients.

## Conclusion

In overall, THRIVE manifested better effectiveness representing as improved oxygenation, prolonged safe apnoea time and decreased risk of complications compared to standard FMV in surgical patients. Therefore, THRIVE could be served as a novel and valuable oxygenation technology for patients during anesthesia induction. However, more high-quality RCTs with big sample sizes are still needed to further testify above findings.

## Supporting information

S1 Checklist PRISMA 2020 checklist. (DOCX)
